# Synthesis, purification, characterization, and antinociceptive activity of opioids peptides

**DOI:** 10.1186/1753-6561-8-S4-P184

**Published:** 2014-10-01

**Authors:** Mariana Nóbrega, Felipe Vinecky, Karla Moreira, Marcia Mortari, Carlos Bloch

**Affiliations:** 1 Programa de Pós-graduação em Biologia Molecular, Universidade de Brasília, DF, Brazil; 2 Laboratório de toxinologia, departamento de fisiologia, instituto de biologia, Universidade de Brasília, DF, Brazil; 3 Embrapa Recursos Genéticos e Biotecnologia, CF, Brazil

**Keywords:** Synthetic peptides, opioids and in vivo tests

## Background

Bioactive peptides prospection is important for biotechnology field as well as a starting point in many research areas, such as: new drugs development and production of genetically modified plants [1]. In general, bioactive peptides have been identified as candidates for the new drugs development because their intrinsic properties concerning some potential activities, like high specificity, potency, less toxicity, and also chemistry and biological diversity [2]. Peptides can present diverse activities such as antimicrobial, opioids, hypotensive, antithrombotic among others. In 2010, around 60 synthetic peptides with therapeutic potential have been available to pharmaceutical marketing and can be used in many pathologies like, allergies, asthma, arthritis, cardiovascular diseases, diabetes, gastrointestinal dysfunction, growth problems, inflammation, obesity, infectious diseases, cancer, osteoporosis, pain, vaccines and others. [3,4]. For pharmaceutical industry, in therapeutic area, the most promising projects are those that focus on cancer, pain, diabetes, Alzheimer's disease and depression [5]. This study aims to design sequences of synthetic peptides that may have dual activity: antinociceptive and hypotensive. These peptides act on different targets, opioids receptors and angiotensin converting enzyme (ACE), respectively.

## Methods

Peptides primary structure prediction was based on previous work of our group, which focused on prospection and bioactive peptides characterization. These studies were based on the knowledge available on the literature about the biological properties of specific protein domains, molecular targets and drug actions. The peptides were synthesized by solid-phase chemical synthesis using Fmoc strategy followed by purification on high performance liquid chromatography. Purity and confirmation of the primary structure were determined by mass spectrometry, MALDI and ESI. The peptides were tested in mice (6-8 per group) intraperitoneally, equimolar to morphine (positive control) in order to assess its possible antinociceptive activity through hot plate and tail flick assays.

## Results and conclusions

It was possible to evaluate the antinociceptive activity of these two designed peptides, which present unpublished sequences. Both exhibited pharmacological activity *in vivo*, which may be denominated opioid receptor agonists, because they promoted antinociception in mice when exposed to thermal stimulation. In naloxone presence, there was no activity, so it can be concluded that the peptides interact with opioid receptors. *In vivo *tests showed that synthetic peptides have delayed response, longer activity compared to morphine and may have cross the blood-brain barrier. Thus, it is interesting continue studying therapeutic potential of these peptides to contribute with development for new drug candidates.
